# MicroRNA-29a-5p Is a Novel Predictor for Early Recurrence of Hepatitis B Virus-Related Hepatocellular Carcinoma after Surgical Resection

**DOI:** 10.1371/journal.pone.0052393

**Published:** 2012-12-20

**Authors:** Hai-Tao Zhu, Qiong-Zhu Dong, Yuan-Yuan Sheng, Jin-Wang Wei, Guan Wang, Hai-Jun Zhou, Ning Ren, Hu-Liang Jia, Qing-Hai Ye, Lun-Xiu Qin

**Affiliations:** Liver Cancer Institute and Zhongshan Hospital, Institutes of Biomedical Sciences, Fudan University, Shanghai, China, Key Laboratory of Carcinogenesis and Cancer Invasion, Ministry of Education, China; Mount Sinai School of Medicine, United States of America

## Abstract

It is still difficult to predict the probability of tumor recurrence after resection of hepatocellular carcinoma (HCC). In this study, we set out to identify specific microRNA (miRNA) in microdissected hepatitis B virus (HBV)-related HCC tissue from formalin-fixed paraffin-embedded (FFPE) samples which might be used in predicting early recurrence after HCC resection. Taqman low density arrays were used to detect the 667 miRNA profiles in both the microdissected tumorous and adjacent non-tumorous liver tissues from 20 HCC patients (discovery set) including 10 patients with early tumor recurrence and 10 without early tumor recurrence and to identify the differentially expressed miRNAs related to HCC recurrence. Then quantitative real-time PCR (qRT-PCR) was used to verify the findings in 106 patients (training set), and to develop a predictive assay. The identified miRNAs were further validated in an independent cohort of 112 patients (validation set). Thirty seven miRNAs were identified from 20 HCC patients and validated in 106 HCC patients using qRT-PCR. A significant association was found between miR-29a-5p level in HCC tissues and early tumor recurrence (P = 0.0002). This association was further confirmed in the independent validation set of 112 patients (P = 0.0154). MiR-29a-5p level was significantly associated with both time to tumor recurrence (TTR) (P = 0.0015) and overall survival (OS) (P = 0.0079) in validation set. In the multivariate analyses, miR-29a-5p was identified as an independent factor for TTR, particularly for those patients with early stage of HCC. The sensitivity and specificity of miR-29a-5p for the prediction of early HCC recurrence of BCLC 0/A stage HCC were 74.2% and 68.2%, respectively. These suggest that miR-29a-5p might be a useful marker for the prediction of early tumor recurrence after HCC resection, especially in BCLC 0/A stage HCCs.

## Introduction

Hepatocellular carcinoma (HCC) is one of the most common and aggressive human cancers worldwide [1]. Hepatic resection remains one of the major curative treatments for HCC [2]. However, its prognosis still remains extremely dismal, which is mainly attributed to the high frequency of intrahepatic metastatic recurrence [3]. Unfortunately, it is still a great challenge to identify the patients who are at a greater risk for tumor recurrence after curative treatment for HCC, particularly in those with early stage disease who do not have significant vascular invasion, or regional lymph node or distant metastasis.

HCC recurrences derived from residual intrahepatic metastasis or multicentric carcinogenesis. Based on the occurring time after HCC treatments, tumor recurrences are divided into early and late recurrence. Usually, early recurrence is believed to occur within two years after HCC treatment and is mainly attributed to the intrahepatic dissemination of metastatic HCC cells; in contrast, late recurrence is thought to originate de novo in at risk liver [4]. These two different kinds of HCC recurrence may need different preventative strategies.

MicroRNAs (MiRNA) are single-stranded RNA molecules of about 21∼23 nucleotides in length, which play an important role in the regulation of gene expression. They are found to be involved in a variety of biological and pathological processes. MiRNAs are the potential biomarkers for cancer, and their profiling may provide more accurate classification of cancer subtypes comparing with the expression profiles of protein-coding genes [5]–[8]. In addition, miRNAs are well preserved in formalin-fixed paraffin-embedded tissue, which makes them ideal candidates for tissue microdissection by which we can acquire a population of “pure” cells for quantitative PCR analyses [9]–[11]. And that is very important for appropriate interpretation of the results.

MiRNAs expression levels have been shown to be useful for predicting recurrence of cancers [12]–[14]. Our previous study also demonstrated a 20-miRNA signature assisting in the identification of HCC patients who are likely to develop metastatic recurrence [15]. In this study, we expected to further identify individual miRNAs that could be used to predict early recurrence of HBV-related HCC after surgical treatment.

## Patients and Methods

A total of 266 patients who underwent curative liver resection for HCC in Zhongshan Hospital, Fudan University (Shanghai, China) from January 2004 to December 2008 were enrolled in the study. Participants provided their written informed consent before participation, and the study protocol was approved by the Institutional Review Board of the Liver Cancer Institute and Zhongshan Hospital, Fudan University. The present clinical investigation has been conducted according to the principles expressed in the Declaration of Helsinki. These patients were divided into four cohorts; the first cohort includes 48 patients that were enrolled in determination of suitable reference genes (Table 1); Another 218 patients were enrolled in identifying the miRNA, which could be used to predict the early recurrence of HCC. These patients were randomly divided into the training (n = 106) and the validation (n = 112) cohort (Table 2). In the training cohort, twenty HCC patients (discovery set) including 10 patients with early tumor recurrence and 10 without early tumor recurrence were enrolled in Taqman low density array analyses, which detected the expression level of 667 miRNAs. There was no significant difference in the clinicopathological features such as tumor stage, the age and gender of patients between the two groups (Table 3). All patients of both training and validation cohorts did not demonstrate any regional lymph node, distant extrahepatic metastasis or macroscopic vascular invasion [3]. Among them, 183 (83.9%) were male. According to the Barcelona Clinic Liver Cancer (BCLC) staging system [16], 142 (65.1%) were Stage 0 or A, the remaining 76 patients (34.9%) were stage B. The diagnosis of HCC was confirmed by two pathologists. No patient received any preoperative anticancer treatment.

**Table 1:** The clinicopathological characteristics of 48 HCC patients enrolled in determination of the suitable reference genes.

	Cohort (n = 48)		*P* value†
Characteristic	Early recurrence (n = 24)	Without early recurrence (n = 24)	
Gender – no. (%)			0.666
Male	22 (91.7)	20 (83.3)	
Female	2 (8.3)	4 (16.7)	
Age – Mean±SD, years	51.2±11.1	48.1±9.8	0.314^‡^
HBV infection – no. (%)	24 (100.0)	24 (100.0)	
HCV infection – no. (%)	0 (0)	0 (0)	
AFP > 200 ng/ml – no. (%)	13 (54.2)	13 (54.2)	1.000
Tumor size – cm			0.118^‡^
Median	5.0	6.5	
Interquartile range	3.0–7.25	4.0–9.5	
Tumor differentiation* – no. (%)			0.039
I–II stage	18 (50.0)	11 (40.0)	
III–IV stage	6 (50.0)	13 (6.0)	
Liver cirrhosis – no. (%)	24 (100.0)	24 (100.0)	1.000
Child–Pugh class A – no. (%)	24 (100.0)	24 (100.0)	1.000
BCLC stage – no. (%)			0.518
0	1 (4.2)	2 (8.3)	
A	11 (45.8)	5 (20.8)	
B	12 (50.0)	17 (70.8)	

The Barcelona Clinic Liver Cancer staging system (BCLC) ranks hepatocellular carcinoma in five stages, ranging from 0 (very early stage) to D (terminal stage).

* Tumor differentiation was assigned by Edmondson's grading system.

† *P* value for the comparison of cohort B with cohort A. ^‡^ Student t tests; Fisher’s exact tests for all the other analyses.

**Table 2:** The clinicopathological characteristics of 218 HCC patients enrolled in this study.

	Cohort (n = 218)		P value†
Characteristics	Training set (n = 106)	Validation set (n = 112)	
Gender – no. (%)			0.854
Male	88 (83.0)	95 (84.8)	
Female	18 (17.0)	17 (15.2)	
Age – Mean±SD, years	52.0±11.3	52.3±10.9	0.834^‡^
HBV infection – no. (%)	106 (100)	112 (100)	
HCV infection – no. (%)	0 (0)	0 (0)	
AFP > 200 ng/ml – no. (%)	42 (39.6)	51 (45.5)	0.378
Tumor size – cm			0.392^‡^
Median	4.4	4.7	
Interquartile range	2.5–5.5	3.0–6.0	
Microvascular invasion – no. (%)	0	0	
Complete tumor encapsulation – no (%)	54 (50.9)	61 (54.5)	0.603
Tumor differentiation* – no. (%)			1.000
I–II stage	81 (76.4)	85 (75.9)	
III–IV stage	25 (23.6)	27 (24.1)	
Liver cirrhosis – no. (%)	91 (85.8)	97 (86.6)	1.000
Child–Pugh class A – no. (%)	99 (95.2)	107 (95.5)	0.489
BCLC stage – no. (%)			0.306
0	19 (17.9)	13 (11.6)	
A	49 (46.2)	61 (54.5)	
B	38 (35.9)	38 (33.9)	
Median follow-up (months)	36.4	40.7	–

The Barcelona Clinic Liver Cancer staging system (BCLC) ranks hepatocellular carcinoma in five stages, ranging from 0 (very early stage) to D (terminal stage).

* Tumor differentiation was assigned by Edmondson's grading system.

† p value for the comparison of cohort B with cohort A. ^‡^ Student t tests; Fisher’s exact tests for all the other analyses.

**Table 3:** The clinicopathological characteristics of 20 HCC patients used in TaqMan low density array.

	Cohort (n = 20)		*P* value†
Characteristic	Early recurrence (n = 10)	Without early recurrence (n = 10)	
Gender – no. (%)			1.000
Male	8 (80.0)	7 (70.0)	
Female	2 (20.0)	3 (30.0)	
Age – Mean±SD, years	51.4±7.6	50.2±11.7	0.789‡
HBV infection – no. (%)	10 (100.0)	10 (100.0)	
HCV infection – no. (%)	0 (0)	0 (0)	
AFP > 200 ng/ml – no. (%)	5 (50.0)	4 (40.0)	1.000
Tumor size – cm			0.753‡
Median	4.0	4.4	
Interquartile range	4.0–5.0	3.6–5.8	
Microvascular invasion – no. (%)	0	0	
Complete tumor encapsulation – no (%)	3(30.0)	4 (40.0)	0.302
Tumor differentiation* – no. (%)			1.000
I–II stage	5 (50.0)	4 (40.0)	
III–IV stage	5 (50.0)	6 (6.0)	
Liver cirrhosis – no. (%)	10 (100.0)	10 (100.0)	1.000
Child–Pugh class A – no. (%)	10 (100.0)	10 (100.0)	0.180
BCLC stage – no. (%)			1.000
0	0	0	
A	8 (80.0)	7 (70.0)	
B	2 (20.0)	3 (30.0)	

The Barcelona Clinic Liver Cancer staging system (BCLC) ranks hepatocellular carcinoma in five stages, ranging from 0 (very early stage) to D (terminal stage).

* Tumor differentiation was assigned by Edmondson's grading system.

† *P* value for the comparison of cohort B with cohort A.

‡ Student t tests; Fisher’s exact tests for all the other analyses.

All patients were followed up till 15 May, 2010, with a median follow-up of 37.2 months (range 3–81 months). During the follow-up, the patients detected the serum α-fetoprotein (AFP) level, ultrasonography and chest X-ray every 2–3 months after operations. Computed tomography (CT) scanning or magnetic resonance imaging (MRI) was done when recurrence was suspected. A diagnosis of tumor recurrence was based on typical appearance on CT scans and/or MRI. A further treatment was given if tumor recurrence was diagnosed according to a uniform guideline as previously described [17].

### Tissue Microdissection and RNA Extraction

The tissue slides of the formalin-fixed paraffin-embedded (FFPE) tissues were reviewed by two experienced pathologists to identify the HCC as well as non-tumorous liver tissues. HCC and non-tumorous liver tissues were microdissected from three representative areas of 12 µm thick tissue sections using the Veritas™ System from Molecular Devices (Arcturus, Mountain View, CA, USA).

RNA extraction was performed according to the protocol of the Recoverall Total Nucleic Acid Isolation Kit (Ambion, Austin, TX, USA). The quantity of total RNA was assessed using the ND-2000 spectrophotometer (NanoDrop Technologies, Wilmington, DE, USA). And the quality of RNA extracted from FFPE samples were assessed by Nanodrop 2000 with A260/A280 around 2.0. The Ct values of the qRT-PCR amplification curves of miR-122 were similar between the snap-frozen and FFPE HCC tissues from the same patient, which also indicated that the quality of RNA obtained from FFPE tissues was adequate and suitable for quantitative miRNA analyses (Figure S1).

### Taqman Low Density Arrays

Taqman low density array (v2.0) is a high throughput PCR-based miRNA array that enables analysis of 667 miRNA assays on microfluidic cards. Each array includes two cards and each card contains 384 assays in which an assay of ath-miR-159a unrelated to any mammalian species is used as negative control. For the initial analysis of miRNAs using the arrays, 10 samples (10 ng each) were pooled together. From each pool, 20 ng of total RNA was added for multiplex reverse transcription reactions. To increase the amount of cDNA available for analysis, a preamplification was carried out. Simultaneous synthesis of cDNA for mature miRNAs was performed using Megaplex reverse transcription primers, Human Pool set 2.0 (Applied Biosystems, Carlsbad, CA, USA), which is a set of pre-defined pools of 671 unique stem-looped miRNA reverse transcription primers. Additionally, each array contains the same four controls. Quantitative real-time PCR (qRT-PCR) was performed using the Applied Biosystems 7900HT Fast Real-Time PCR System, and default thermal-cycling conditions.

### QRT-PCR Validation

QRT-PCR was performed in triplicate with Taqman assay (Applied BioSystems, Foster city, CA), including non-template controls that ensured a lack of signal in the assay background. Twenty nanogram of RNA solution from the 40 µl elute from RNA isolation of a given sample was used as input into the reverse transcription (RT) reaction. Input RNA was reverse transcribed using the Taqman miRNA reverse transcription kit and miRNA-specific stem-loop primers (Applied BioSystems, Foster city, CA) in a small-scale RT reaction (5 µl). Data were analyzed with SDS Relative Quantification Software version 2.3 and RQ manager 1.2 (Applied BioSystems, Foster city, CA), with the automatic cycle threshold (Ct) setting for assigning baseline and threshold for Ct determination.

### Statistical Methods

The endpoints included the time to recurrence (TTR) and overall survival (OS). TTR was defined as the time from the start of surgery to the first report of intrahepatic recurrence (excluding the patients who had died from non-HCC-related causes before tumor recurrence). For patients who had not experienced a recurrence during the follow-up, TTR was censored at the date of death or the last follow-up. OS was defined as the interval between the dates of surgery and death [18].

High throughput data generated from TaqMan low density array were analyzed by RQ manager 1.2. Kaplane-Meier analysis was performed to compare TTR and OS between the different groups of patients using Prism 5.0 software (GraphPad, San Diego, CA, USA) and statistical P values were generated by the Cox-Mantel log-rank test. Univariate and multivariate analyses were based on the Cox proportional hazards regression model. Statistical analysis was performed using SPSS 15.0 (Chicago, IL, USA). Distribution of continuous data was determined using the Kolmogorove-Smirnov Z test. A two-sample t-test was used to compare log 2-transformed Ct values of the candidate reference genes, and log 2-transformed relative quantities of target miRNAs between microdissected tumor and adjacent non-tumorous liver tissues. One way ANOVA was used to compare log 2-transformed Ct values of candidate reference genes among four groups. The equivalence test was used to determine if reference genes were equivalently expressed between groups [19]. The χ2 test or Fisher’s exact test were used to compare qualitative variables. The ROC (receiver operator characteristic) curve was generated to determine the optimal cutoff point of high and low expression of miR-29a-5p. P value <0.05 was considered significant. The expression of target miRNA was determined by the delta Ct comparative threshold (ΔCt) method (ΔCt = Ct target gene-Ct reference gene).

## Results

### Determination of Suitable Reference Genes

MiRNAs are well preserved in formalin-fixed paraffin-embedded tissue and are suitable for tissue microdissection. However, no systematic study on suitable reference genes for qRT-PCR analysis in microdissected miRNA from HCC FFPE samples has been reported. In order to determine the suitable reference genes in this study, we applied the established approach of finding miRNAs whose expression patterns were similar to the global mean expression of Taqman low density array [20]. MiR-374a, miR-379, miR-889 were selected from card A. MiR-769-5p, miR-26a-1*, miR-625* were selected from card B. Besides, U6, RNU6B, let-7a, miR-191, miR-103, miR-328, miR-25 were chosen for further validation in a larger cohort because they were widely used or recommended as reference genes in HCC or solid cancer miRNA studies (Table S1) [21]–[27]. This cohort was composed of 48 samples comprised both microdissected tumorous and adjacent non-tumorous liver tissues (Table 1). Among which, 24 HCC samples were with early recurrence, while another 24 samples were without early recurrence. Mir-26a-1* and miR-889 cannot be detected in all samples, so they were excluded from further validation. The stabilities of the reference genes were assessed by geNorm and NormFinder algorithms after confirmation of their equivalent expression between groups (Figure S2, S3). Finally, we identified the combination of miR-103 and miR-191 was the most stable reference genes for miRNA analysis of microdissected HCC and adjacent non-tumorous liver tissues (Figure S4, Table S2).

### Identification of miRNAs that Associated with Early HCC Recurrence after Liver Resection

Usually early recurrence is believed to occur within two years after HCC treatment mainly contributed by dissemination of metastatic HCC cells; in contrast, late recurrence is thought to originate de novo in at risk liver [28], [29]. We first analyzed the cumulative recurrence in all 218 patients, and found that tumor recurrences were biphasic (Figure S5), one happened within 24 months after HCC resection (early recurrence), another was 48–72 months (late recurrence).

After Taqman low density array analysis, a total of 37 miRNAs that satisfied the following two criteria were thought to be related to HCC recurrence and selected for further qRT-PCR validation: (i) the Ct value was below 32 in at least one group; (ii) the difference between the above two groups was more than 1 or less than -1 (Table S3-S5). Among them, 19 miRNAs were found to be upregulated in microdissected HCC tissue with early recurrence (miR-29a-5p, miR-27b*, miR-204, miR-29c, miR-10b, miR-196b, miR-216a, miR-217, miR-517a, miR-518e, miR-518f, miR-518b, miR-519a, miR-519d, miR-522, miR-486-5p, miR-181c, miR-210, miR-215); 11 miRNAs were found to be down-regulated in microdissected HCC tissue with early recurrence (miR-193b*, miR-643, miR-22, miR-15b, miR-505, miR-107, miR-142-5p, miR-135a, miR-34c-5p, miR-98, miR-483-5p); eight miRNAs were found to be upregulated in microdissected non-tumorous liver tissue with early recurrence (miR-486-5p, miR-181c, miR-193b*, miR-643, miR-409-3p, miR-424*, miR-139-3p, miR-766); six miRNAs were found to be down-regulated in microdissected non-tumorous liver tissue with early recurrence (miR-210, miR-215, miR-22, miR-409-5p, miR-200a*, miR-10b*).

### Identification of Prognostic miRNAs from Training Set and Verification in an Independent Validation Set

**Figure 1:**
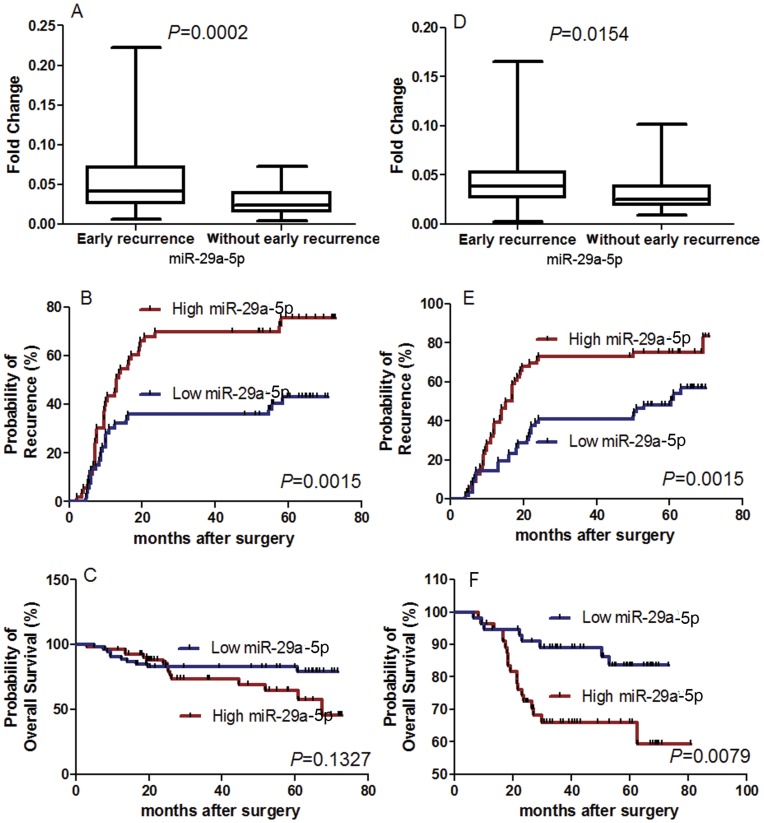
The association of miR-29a-5p level in HCC tissues with early tumor recurrence after HCC resection. Box and whiskers plots show the expression levels of miR-29a-5p between early recurrence and without early recurrence group in training (A) and validation (D) set; the miR-29a-5p level in HCC tissues of early recurrence group was obviously higher than those without recurrence. Kaplan Meier curves showed the association of miR-29a-5p in HCCs with time to recurrence (TTR) (B, E) and overall survival (OS) (C, F) in the training (B, C) and validation (E, F) sets. The high- and low miR-29-5p subgroups were divided using the median level of miR-29a-5p in the training set as the cut-off value. The miR-29a-5p level was significantly associated with TTR in both the training (B) and validation (E) sets; and OS in the validation set (F), but not in the training set (C).

The association of the selected miRNAs in microdissected HCC tissues with HCC recurrence was further validated in 106 HCC patients (training set) using qRT-PCR. Among them, only the miR-29a-5p level in microdissected HCC tissues was demonstrated to be significantly associated with early HCC recurrence. The miR-29a-5p level in HCC tissues from the patients with early tumor recurrence was obviously higher than those without recurrence (P = 0.0002) (Figure 1A). In addition, the associations of miRNAs selected above with OS and TTR were investigated in 106 HCC patients of the training set. In the Kaplan–Meier analyses, the miR-29a-5p level was significantly associated with TTR (P = 0.0015) (Figure 1B). The patients with higher expression of miR-29a-5p exhibited a shorter TTR compared those with low expression of miR-29a-5p; however, the association between miR-29a-5p level and OS didn’t reach significant (P = 0.1327) (Figure 1C).

To further validate the prognostic role of miR-29a-5p, we analyzed its expression level with HCC prognosis in the independent validation cohort of 112 patients (validation set). Similarly, the significant correlation between the higher miR-29a-5p levels in tumor tissues and a higher possibility of early tumor recurrence after operation was further confirmed (P = 0.0154) (Figure 1D). Moreover, the miR-29a-5p level was found to be significantly associated with TTR (P = 0.0015) (Figure 1E) as well as OS (P = 0.0079) (Figure 1 F) of HCC patients. The patients in the high expression of miR-29a-5p group had a significantly shorter TTR and OS than those in the low expression of miR-29a-5p group.

### Univariate and Multivariate Analyses of the Prognostic Abilities of miR-29a-5p

To further evaluate the prognostic value of miR-29a-5p for HCC, univariate and multivariate analyses were performed with the clinicopathological characteristics and the expression of miR-29a-5p in all patients enrolled in this study. In univariate analysis, the expression level of miR-29a-5p, tumor numbers, and Okuda stage were significantly associated with TTR. In multivariate analysis, the expression of miR-29a-5p was still revealed to be independent prognostic indicator for TTR (P = 0.004) (Table 4).

**Table 4:** Univariate and multivariate Cox regression analyses of miR-29a-5p for TTR and OS of HCC patients.

	TTR		OS	
	Hazard ratio		Hazard ratio	
Variables	(95% CI)*	*P* Value	(95% CI)*	*P* Value
**Univariate analysis**				
miR-29a-5p (low vs high)	0.5 (0.3 to 0.8)	0.003	1.0 (0.7 to 1.4)	0.901
Gender (male vs female)	1.2 (0.6 to 2.4)	0.632	0.7 (0.4 to 1.1)	0.164
Age (>50 vs ≤50)	1.2 (0.7 to 2.1)	0.415	1.1 (0.8 to 1.6)	0.553
BCLC stage (B vs 0 and A)	1.3 (0.8 to 2.1)	0.374	1.1 (0.8 to 1.6)	0.446
Okuda stage (I vs II)	0.4 (0.2 to 1.0)	0.046	1.1 (0.7 to 1.7)	0.620
CLIP stage (2+3 vs 0+1)	1.1 (0.7 to 1.8)	0.687	1.2 (0.8 to 1.6)	0.411
Tumor size (>5cm vs ≤5cm)	0.8 (0.5 to 1.4)	0.458	1.2 (0.9 to 1.8)	0.246
Tumor encapsulation (complete vs none)	1.5 (0.9 to 2.4)	0.138	1.0 (0.7 to 1.4)	0.892
Tumor differentiation (III–IV vs I–II)	1.3 (0.7 to 2.3)	0.375	1.0 (0.7 to 1.5)	0.840
Tumor number (multiple vs single)	1.6 (1.0 to 2.6)	0.037	1.2 (0.9 to 1.7)	0.256
AFP (>200 ng/ml vs ≤200 ng/ml)	1.0 (1.0 to 1.0)	0.989	1.2 (0.8 to 1.6)	0.359
**Multivariate analysis**				
miR-29a-5p (low vs high)	0.5 (0.3 to 0.8)	0.004		
Okuda stage (I vs II)	0.4 (0.2 to 1.0)	0.066		
Tumor number (multiple vs single )	1.6 (1.0 to 2.6)	0.037		

The Barcelona Clinic Liver Cancer staging system (BCLC) ranks hepatocellular carcinoma in five stages, ranging from 0 (very early stage) to D (terminal stage).

* Tumor differentiation was assigned by Edmondson's grading system.

† *P* value for the comparison of cohort B with cohort A. ^‡^ Student t tests; Fisher’s exact tests for all the other analyses.

In this study, we excluded those patients with macroscopic or microscopic vascular invasion which is thought to be a major risk factor for disease free survival.

### The Prognostic Value of miR-29a-5p Level for Tumor Recurrence in Patients with Early Stages of HCC

**Figure 2:**
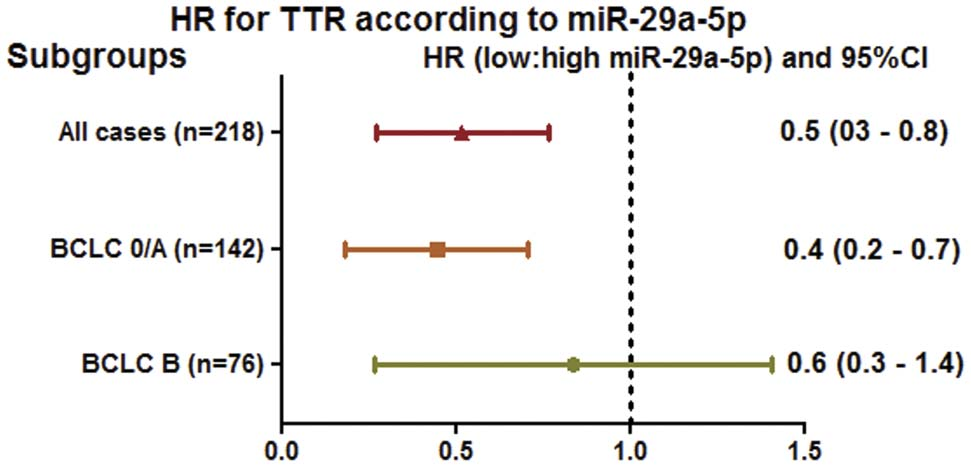
Hazard ratios (HRs) of miR-29a-5p levels for time to recurrence (TTR) in different BCLC stages of HCC patients. The HRs of miR-29a-5p levels for TTR in each subgroup of patients with HCC were calculated by comparing the patients with low miR-29a-5p with those with high miR-29a-5p level. HR <1.0 indicates a worse outcome for those with a higher miR-29a-5p level. The miR-29a-5p levels were significantly associated with TTR in all patients, and particularly those with BCLC 0/A stage of HCC. BCLC, Barcelona Clinic Liver Cancer staging system.

We further evaluated the prognostic value of miR-29a-5p for specified subgroups of patients according to BCLC staging system. Interestingly, the expression level of miR-29a-5p were found to be significantly associated with TTR in all patients and those with BCLC 0/A, but not BCLC B stage of HCC (Table 5, Figure 2).

**Table 5:** Univariate Cox regression analyses of miR-29a-5p for TTR of patients with different stages of HCCs.

	TTR	
	Hazard ratio	
Variables	(95% CI)*	*P* Value
**Univariate analysis**		
**BCLC 0/A stage**		
miR-29a-5p (low vs high)	0.4 (0.2 to 0.7)	0.003
Gender (male vs female)	0.9 (0.4 to 2.2)	0.865
Age (>50 vs ≤50)	1.4 (0.7 to 2.7)	0.315
Tumor encapsulation(none vs complete)	1.3 (0.7 to 2.5)	0.411
Tumor differentiation (III–IV vs I–II)	1.9 (0.9 to 3.7)	0.071
AFP (>200 ng/ml vs ≤200 ng/ml)	0.8 (0.4 to 1.6)	0.507
**BCLC B stage**		
miR-29a-5p (low vs high)	0.8 (0.6 to 1.2)	0.286
Gender (male vs female)	1.8 (0.5 to 6.0)	0.359
Age (>50 vs ≤50)	1.0 (0.4 to 2.3)	0.990
Tumor encapsulation(none vs complete)	1.7 (0.7 to 4.0)	0.210
Tumor differentiation (III–IV vs I–II)	0.7 (0.3 to 2.0)	0.563
AFP (>200 ng/ml vs ≤200ng/ml)	0.7 (0.3 to 1.5)	0.359

The Barcelona Clinic Liver Cancer staging system (BCLC) ranks hepatocellular carcinoma in five stages, ranging from 0 (very early stage) to D (terminal stage).

* Tumor differentiation was assigned by Edmondson's grading system.

† *P* value for the comparison of cohort B with cohort A. ^‡^ Student t tests; Fisher’s exact tests for all the other analyses.

Of the 142 patients at stage 0/A, the early recurrence rate was 54.2%. Among these patients, 69 were identified as having high level of miR-29a-5p and 73 were identified as having low miR-29a-5p in their tumors. Patients with high miR-29a-5p had a higher early recurrence rates in contrast to those with low miR-29a-5p (73.9% versus 35.6%; P<0.001).

Of the 76 BCLC stage B patients, the early recurrence rate of patients with high level of miR-29-5p (n = 34) was also higher than those with low miR-29a-5p (n = 42) (61.8% versus 47.6%, P = 0.219), although the comparison did not show statistical significance.

**Figure 3:**
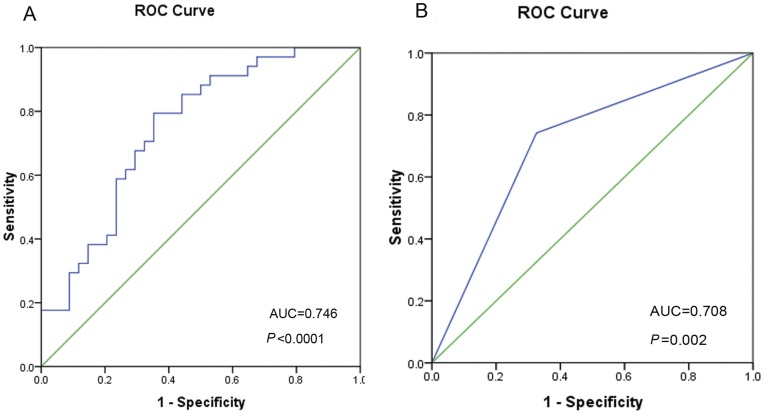
The receiver operating characteristic (ROC) curve analyses of the sensitivity and specificity of miR-29a-5p level in HCCs for the prediction of early HCC recurrence. In the training set (A), the area under curve (AUC) was 0.746 and the optimal cutoff of miR-29a-5p (ΔCt = 4.85) was determined, the AUC of the validation set (B) was 0.708.

The receiver operating characteristic (ROC) curve was plotted to identify a cut-off value and evaluate the sensitivity and specificity of miR-29a-5p for early recurrence of BCLC 0/A stage HCC. Using the optimal cut-off value (ΔCt = 4.85) which was determined by ROC curve in the training cohort, the sensitivity and specificity of miR-29a-5p were 70.6% and 67.6%, respectively, with an AUC of 0.746 in the training set (Figure 3A); while in the validation set, the sensitivity and specificity were 74.2% and 68.2%, respectively, with an AUC of 0.708 (Figure 3B). However, we did not find any significant association between the expression level of miR-29a-3p, the counterpart of miR-29a-5p, in HCC tissues and early tumor recurrence after HCC resection (Figure S6).

### Association between the Expression Levels of Selected miRNAs in Microdissected non-tumorous Liver Tissues and Early Recurrence of HCC

The association of the selected miRNAs in microdissected non-tumorous liver tissues with HCC recurrence was further validated in both training set and validation set using qRT-PCR. We failed to detect any miRNA in the non-tumorous liver tissues which was significantly correlated to the TTR or OS of HCC patients in both sets of patients.

## Discussion

Currently, some clinical and pathological features, such as tumor vascular invasion and tumor stage, are used to predict the early recurrence of HCC, and several staging systems are available for stratifying HCC patients [30]–[33]. However, they have limitations in determining clinical outcome, especially in patients with early stage disease without vascular invasion [33]. Identification of molecular markers may provide supplemental and useful information for predicting clinical outcome in patients with a given stage of disease and improve the selection of patients for adjuvant therapies after resection [34]. Such a molecular system for HCC has yet to be established, although some preliminary molecular signatures coming from the tumor or its microenvironment have the capacity to discriminate subgroups with different outcomes [15], [35]–[38]. MiRNAs, small non-coding RNAs, have been demonstrated to be deregulated, and involved in the initiation and progression of human cancers including HCC, and could be potential biomarkers for diagnosis and prognosis [39]. In this study, we focused on the prognostic values of miRNA(s) for HCC patients, including those with early stage HCCs.

Specific genetic fingerprints are being established for many diseases which are useful for diagnosis and prognosis and as a guide to therapy. However, even the most sophisticated genetic validation methods will be of limited value if the input DNA, RNA, or proteins are not derived from pure populations of cells exhibiting the characteristic disease morphology. Microdissection can address the problems associated with analysis of heterogeneous tissue [11]. The gene-expression profiling of microdissected tissues has been used for molecular classification and prognostication of cancer patients, however, its application is hindered by technical limitations – in particular, the requirement of frozen samples because frozen samples are not banked in hospital currently, and that means frozen samples available for analysis are very limited and with short follow-up. Fortunately, researches have found that the formalin-fixed paraffin-embedded (FFPE) tissues are suitable for miRNA investigation [9], [10]. These paved the way to evaluate the prognosis of HCC patients by tissue microdissection of FFPE samples and high throughput analysis. Alterations of miRNA profiling have been investigated in various kinds of human cancers, and identified distinct expression profiles for each cancer. And more, these cancer-specific miRNA expression profiles were able to be used to identify poorly differentiated tumors. Thus, miRNA profiling appears to be a potentially useful tool in cancer diagnosis and classification [5]. Moreover, many miRNAs have also been demonstrated to involve in cancer metastasis and can be used to predict cancer prognosis. MiR-1 might serve as a prognostic marker for human prostate cancer [40]. Upregulation of miR-155 was found to promote cancer cell invasion and predict poor survival of HCC after liver transplantation [41]. MiR-221 was associated with the prognosis of prostate cancer and HCC [42], [43].

In this study, through detecting the miRNA profiles in a large cohort of HCC specimens, we identified 37 miRNAs that might be related to HCC recurrence by Taqman low density arrays. Among of them, miR-29a-5p level in HCC tissues was demonstrated to be significantly associated with early HCC recurrence after operation, including those early stage HCCs. The significant correlation between the higher miR-29a-5p levels in HCCs and a higher possibility of early recurrence after operation was further confirmed in an independent validation set of 112 patients. Its sensitivity and specificity could reach to around 70% for those HCC patients with BCLC 0/A stage. According to BCLC staging system, stage 0 and A are considered the early stage, which means these HCC patients may have better prognosis, including longer time to recurrence and better overall survival rate. However, some still have poor prognosis in clinical practice, and existing prognostic factors are less informative. It is a major challenge in the prognostic prediction of these patients for clinicians. In our study, the expression level of miR-29a-5p may help to discriminate them from other early-stage patients. And in multivariate analysis, the prognostic significance of miR-29a-5p was independent of other clinicopathological features of HCC patients. These suggest that miR-29a-5p could serve as a novel predictor for early tumor recurrence after HCC resection.

Many miR-29 family members were found to be dysregulated and associated with the prognosis of cancers. The miR-29a-3p levels in both serum and tumor tissues have been demonstrated to be significantly higher in colorectal liver metastasis (CRLM) patients compared with in colorectal cancer (CRC) patients [44]. And miR-29 family (miR-29a-3p/b/c) was found to regulate specific genes that associated with tissue invasiveness and metastasis in lung adenocarcinoma [45]. In present study, we found that high level of miR-29a-5p was correlated with the metastasis-related early tumor recurrence of HCC, which is consistent with the above reports. However, in another study about HCC by Xiong et al., miR-29 family (miR-29a-3p/b/c) was found to be down-regulated in HCC tissue and may have a potential tumor suppressive effect. Moreover, lower expression of miR-29b was found to be correlated with shorter disease-free survival of HCC patients [46]. The real reason of these contradictory results is not clear, which might attribute to the different sequences and target genes of different miR-29 family members tested, and deserves further validation.

The mechanism of miR-29a-5p involved in early recurrence of HCC is not clear yet, it might play an important role in HCC invasion and metastasis which is the main reason for early tumor recurrence after HCC resection [4], [31], [37]. Unraveling the possible role of miR-29a-5p in metastatic recurrence of HCC may be helpful in the development of new therapeutic strategy for patients with HCC.

There have been many reports about the roles of miR-29a-3p, the counterpart of miR-29a-5p, in the development and progression of cancers. MiR-29a-3p and miR-29a-5p present only partial complementary overlap and have different spectrums of targets although produced from the same primary transcript. MiR-29a-3p has been demonstrated to regulate long non-coding RNA gene MEG3 in HCC and contribute to HCC growth [47]; and overexpression of miR-29a-3p promotes migration of hepatoma cells [46], [48]. However, in this study, we did not find any significant association between the miR-29a-3p level in HCC tissues and early tumor recurrence after HCC resection.

It has been reported that the gene profiling of non-tumor liver tissues can be used to predict HCC recurrence, particular for late tumor recurrence after operation which is thought to mainly originate de novo in at risk liver [37]. In this study, we have also detected the miRNA expression profiles in the non-tumorous liver tissues; however, we did not find any of which was significantly correlated to early tumor recurrence or survival of HCC patients.

In summary, based on the investigation of two independent large cohorts of patients with long-term follow-up, we found that the expression level of miR-29a-5p in HCC FFPE tissues could provide useful information for predicting early recurrence after HCC resection including early stage (BCLC 0/A) of HCC. This finding may potentially enable us to identify and select high-risk patients for effective adjuvant therapy.

## Supporting Information

Figure S1 **MicroRNA accessibility in snap-frozen versus FFPE HCC tissues.** The qRT-PCR amplification curves of miR-122 in snap-frozen (A) and FFPE HCC tissues (B) from the same patient with the same input total RNA (5 ng, 10 ng, 15 ng, 20 ng) showed similar Ct values, which indicated that the quality of RNA obtained from FFPE tissues was adequate and suitable for quantitative miRNA analyses.
(TIF)

Figure S2 **Expression of candidate reference genes.** (A) Quantity of candidate reference miRNAs and snRNAs in microdissected tumor tissues and adjacent non-tumorous liver tissues as expressed as real-time PCR cycle threshold numbers (Ct values). Boxes represent the lower and upper quartiles with medians. Outliers (values that are between 1.5 and 3 times the interquartile range) are presented by circles beyond the whiskers. Extreme values (values that are more than 3 times the interqurtile range) are depicted with the symbol (★). Two-sample t-test shows no significant difference in all candidate reference genes except miR-625*, miR-769-5p, miR-374a. (B) The variation associated with reference gene expression differed significantly among the candidate reference genes (P<0.001), with miR-379 and U6 showing the greatest variation.
(TIF)

Figure S3 **Expression and equivalence test of candidate reference genes.** (A) Quantity of candidate reference miRNAs and snRNAs in early recurrence microdissected tumor tissues (EC) and adjacent non-tumorous liver tissues (EB), without early recurrence microdissected tumor tissues(LB) and adjacent non-tumorous liver tissues (LB) as expressed as real-time PCR cycle threshold numbers (Ct values). Boxes represent the lower and upper quartiles with medians. Outliers (values that are between 1.5 and 3 times the interquartile range) are presented by circles beyond the whiskers. Extreme values (values that are more than 3 times the interqurtile range) are depicted with the symbol (★). One way ANOVA shows no differential expression of all candidate reference genes among all four groups except miR-625*, miR-769-5p and miR-374a. (B) Equivalence test for candidate reference genes. Each line indicates the difference in logarithmic (base 2) expression level between tumorous tissue and non-tumorous liver tissue groups, with the upper and lower bars representing the upper and lower limits of symmetrical confidence intervals respectively. All genes except miR-374a, miR-625*, miR-769-5p were equivalently expressed with confidence intervals within fold change of 2 (deviation area 1, −1) and including 0.
(TIF)

Figure S4 **Ranking of candidate reference genes with geNorm.** (A) Expression stability of the reference genes as calculated by geNorm. Stability value M is based on the average pair-wise variation between all genes. The least stable gene with highest M value was excluded and M value recalculated till end up with the most stable pair. (B) The GeNorm algorithm calculates a normalization factor (NF) which is used to determine the optimal number of reference genes required for accurate normalization. This factor is calculated using the variable V as the pairwise variation (Vn/Vn+1) between two sequential NFs (NFn and NFn+1). To meet the recommended cut off V-value which is the point at which it is unnecessary to include additional genes in a normalization strategy. The recommended limit for V value is 0.15 but it is not always achievable. In this instance, the GeNorm output file indicated that the optimal number of genes required for normalization was two.
(TIF)

Figure S5 **Cumulative recurrence rate per month over time after HCC resection.** The cumulative recurrence in all 218 patients showed that the HCC recurrence rate is biphasic.
(TIF)

Figure S6 **The association of miR-29a-3p level in microdissected HCC tissues with early tumor recurrence after HCC resection.** Box and whiskers plots demonstrated that there was no significant difference of miR-29a-3p levels in HCC tissues was found between the patients with and without early recurrence in the training set (P = 0.6279).
(TIF)

Table S1 **Details of microRNAs and snRNAs enrolled in determination of suitable reference genes.**
(DOC)

Table S2 **Stability value and ranking of reference genes based on NormFinder.**
(DOC)

Table S3 **Thirty-seven miRNAs identified that might be related to HCC recurrence after operation in this study.**
(DOC)

Table S4 **Results of TaqMan Low-Density Array qRT-PCR (Card A).**
(XLS)

Table S5 **Results of TaqMan Low-Density Array qRT-PCR (Card B).**
(XLS)
