# Supplementary figures and images for: MicroRNA-29a-5p Is a Novel Predictor for Early Recurrence of Hepatitis B Virus-Related Hepatocellular Carcinoma after Surgical Resection
Source: PLoS One. 2012 Dec 20;7(12):e52393. doi: 10.1371/journal.pone.0052393 (PMC3527523; doi:10.1371/journal.pone.0052393)

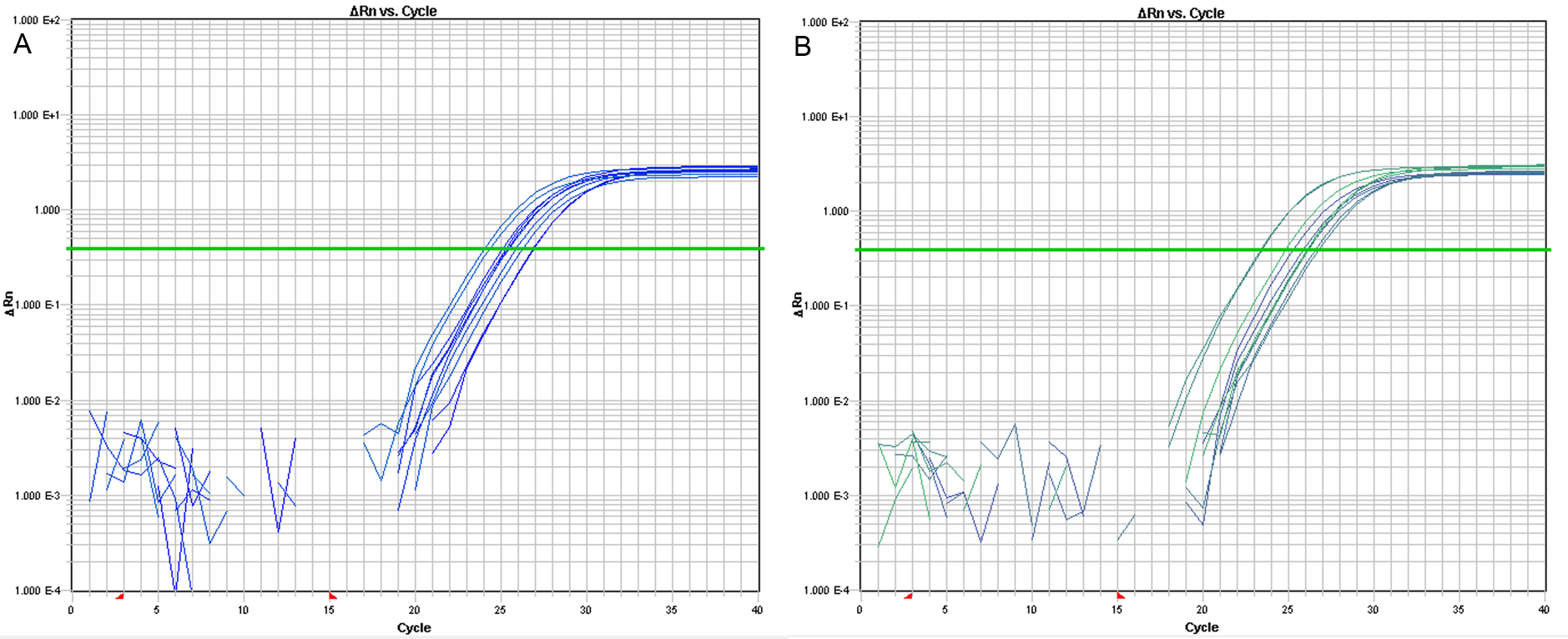

Supplement: Figure S1 — MicroRNA accessibility in snap-frozen versus FFPE HCC tissues. The qRT-PCR amplification curves of miR-122 in snap-frozen (A) and FFPE HCC tissues (B) from the same patient with the same input total RNA (5 ng, 10 ng, 15 ng, 20 ng) showed similar Ct values, which indicated that the quality of RNA obtained from FFPE tissues was adequate and suitable for quantitative miRNA analyses. (TIF) [file pone.0052393.s001.tif]

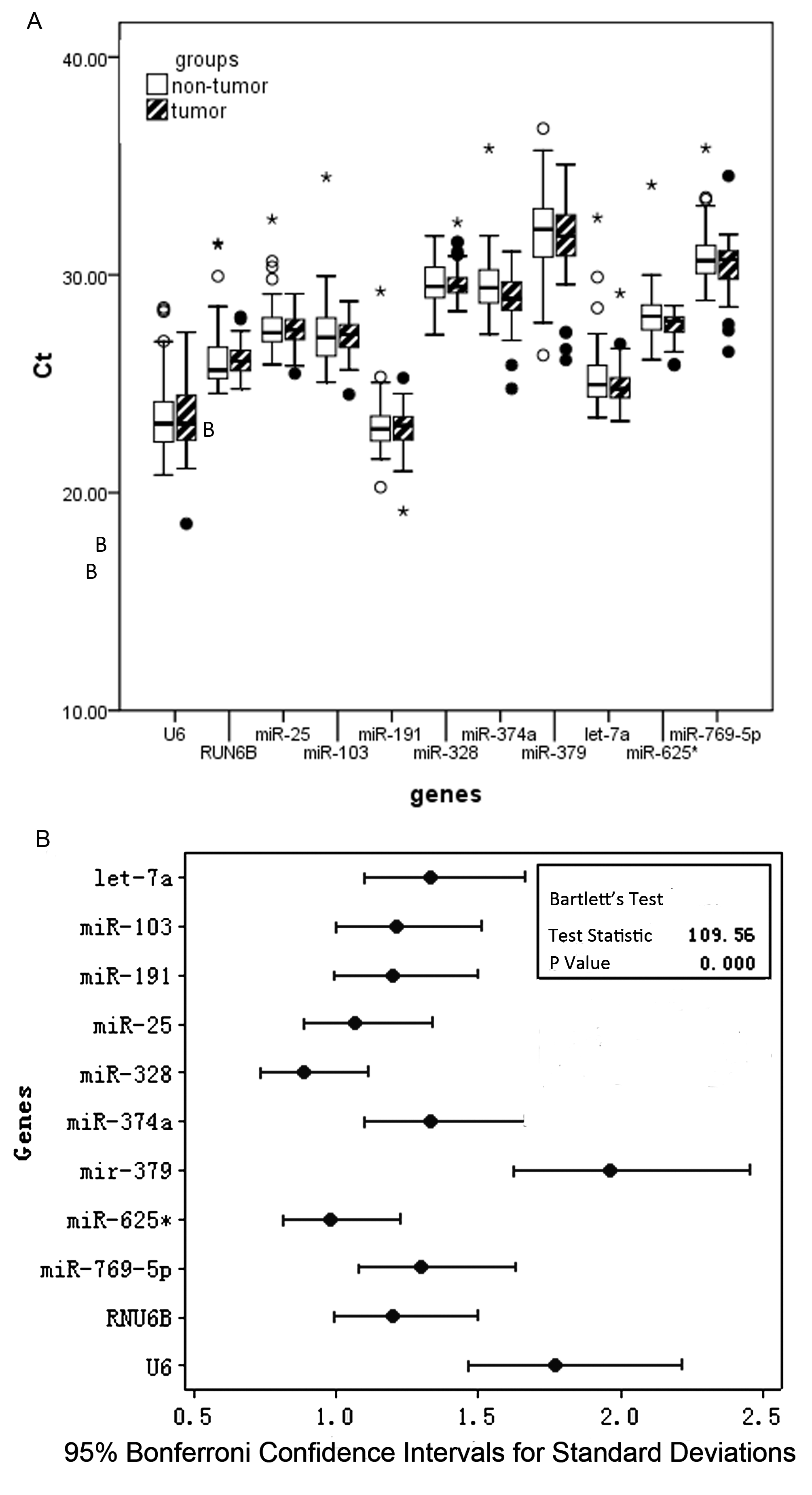

Supplement: Figure S2 — Expression of candidate reference genes. (A) Quantity of candidate reference miRNAs and snRNAs in microdissected tumor tissues and adjacent non-tumorous liver tissues as expressed as real-time PCR cycle threshold numbers (Ct values). Boxes represent the lower and upper quartiles with medians. Outliers (values that are between 1.5 and 3 times the interquartile range) are presented by circles beyond the whiskers. Extreme values (values that are more than 3 times the interqurtile range) are depicted with the symbol (★). Two-sample t-test shows no significant difference in all candidate reference genes except miR-625*, miR-769-5p, miR-374a. (B) The variation associated with reference gene expression differed significantly among the candidate reference genes (P<0.001), with miR-379 and U6 showing the greatest variation. (TIF) [file pone.0052393.s002.tif]

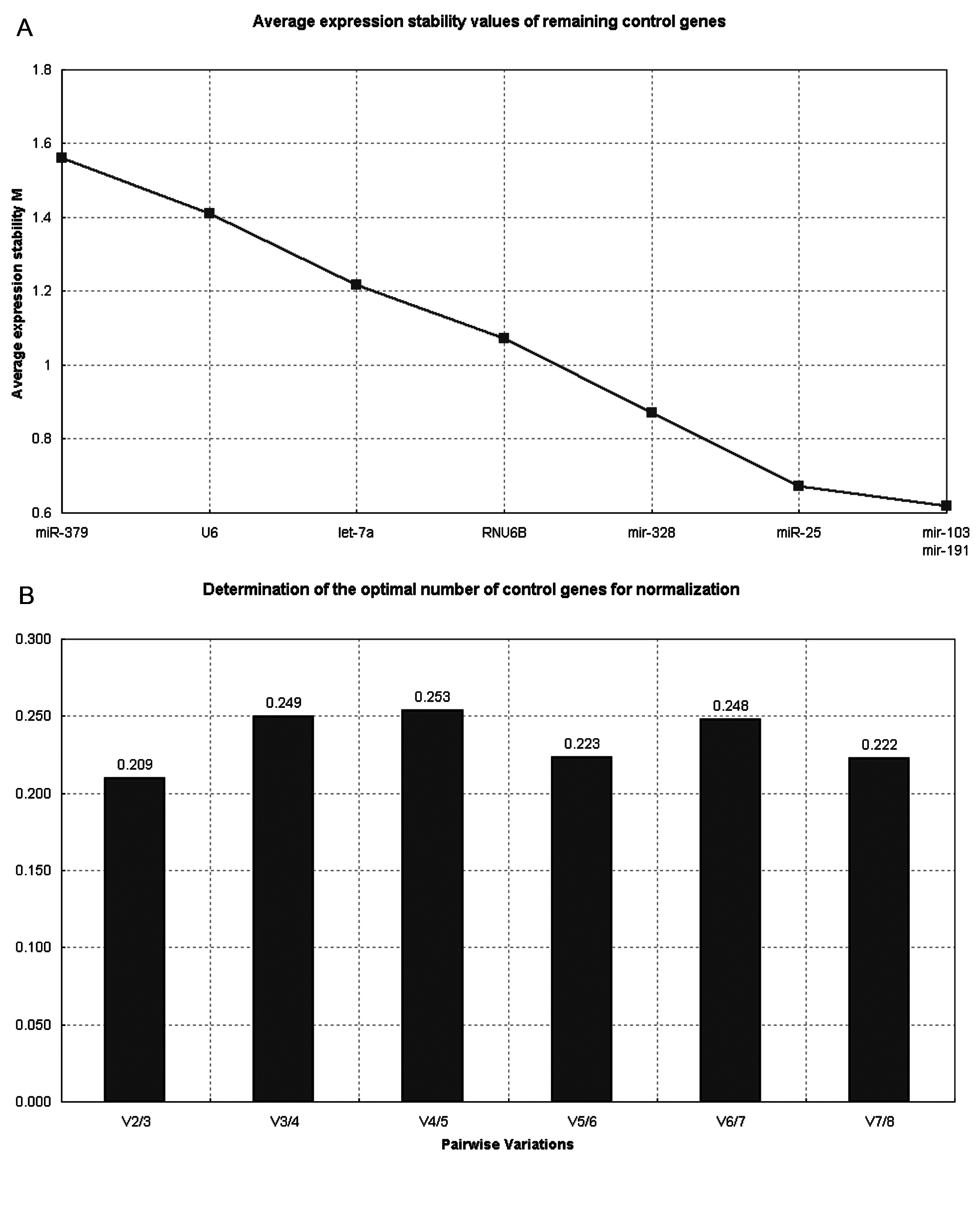

Supplement: Figure S4 — Ranking of candidate reference genes with geNorm. (A) Expression stability of the reference genes as calculated by geNorm. Stability value M is based on the average pair-wise variation between all genes. The least stable gene with highest M value was excluded and M value recalculated till end up with the most stable pair. (B) The GeNorm algorithm calculates a normalization factor (NF) which is used to determine the optimal number of reference genes required for accurate normalization. This factor is calculated using the variable V as the pairwise variation (Vn/Vn+1) between two sequential NFs (NFn and NFn+1). To meet the recommended cut off V-value which is the point at which it is unnecessary to include additional genes in a normalization strategy. The recommended limit for V value is 0.15 but it is not always achievable. In this instance, the GeNorm output file indicated that the optimal number of genes required for normalization was two. (TIF) [file pone.0052393.s004.tif]

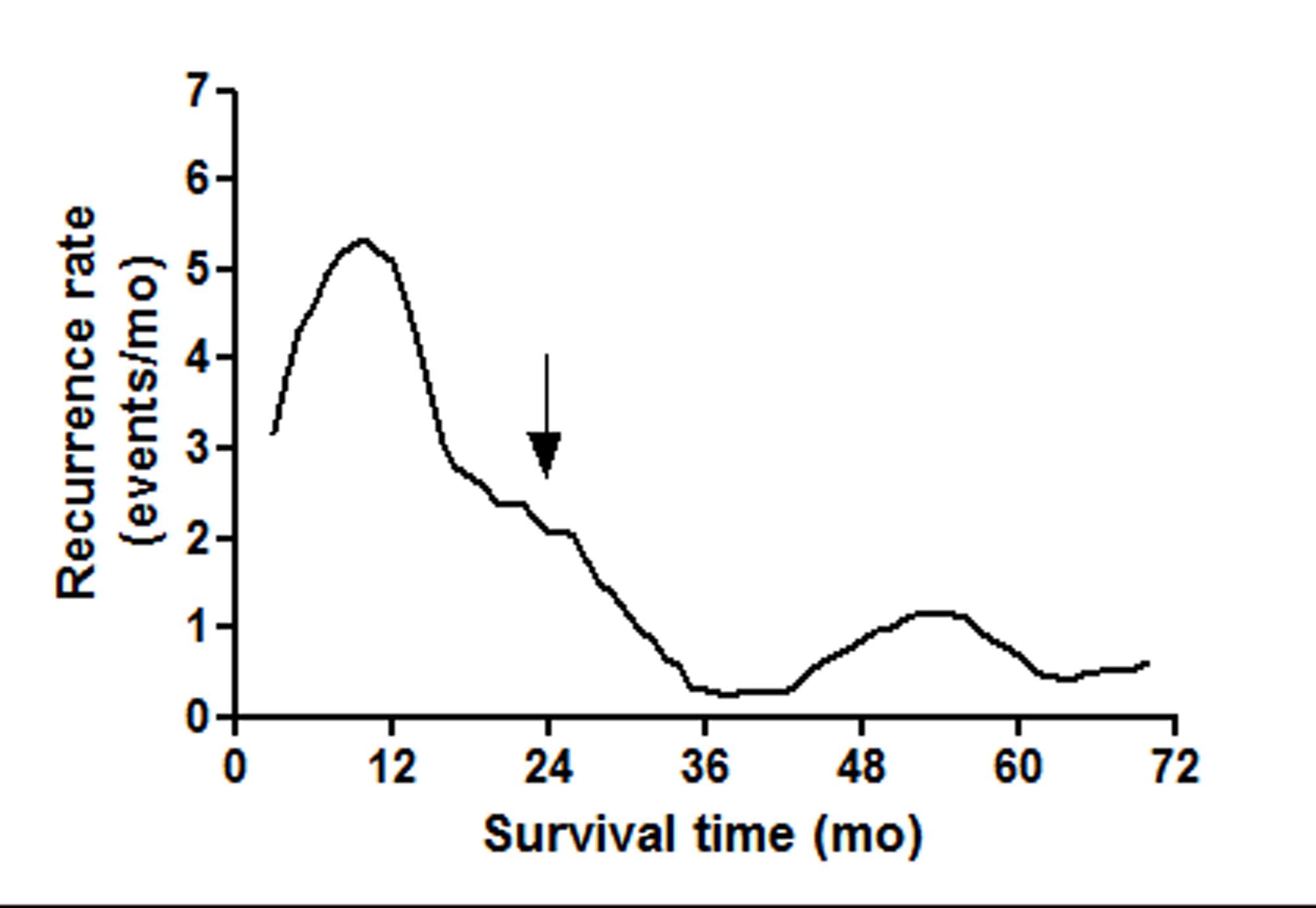

Supplement: Figure S5 — Cumulative recurrence rate per month over time after HCC resection. The cumulative recurrence in all 218 patients showed that the HCC recurrence rate is biphasic. (TIF) [file pone.0052393.s005.tif]

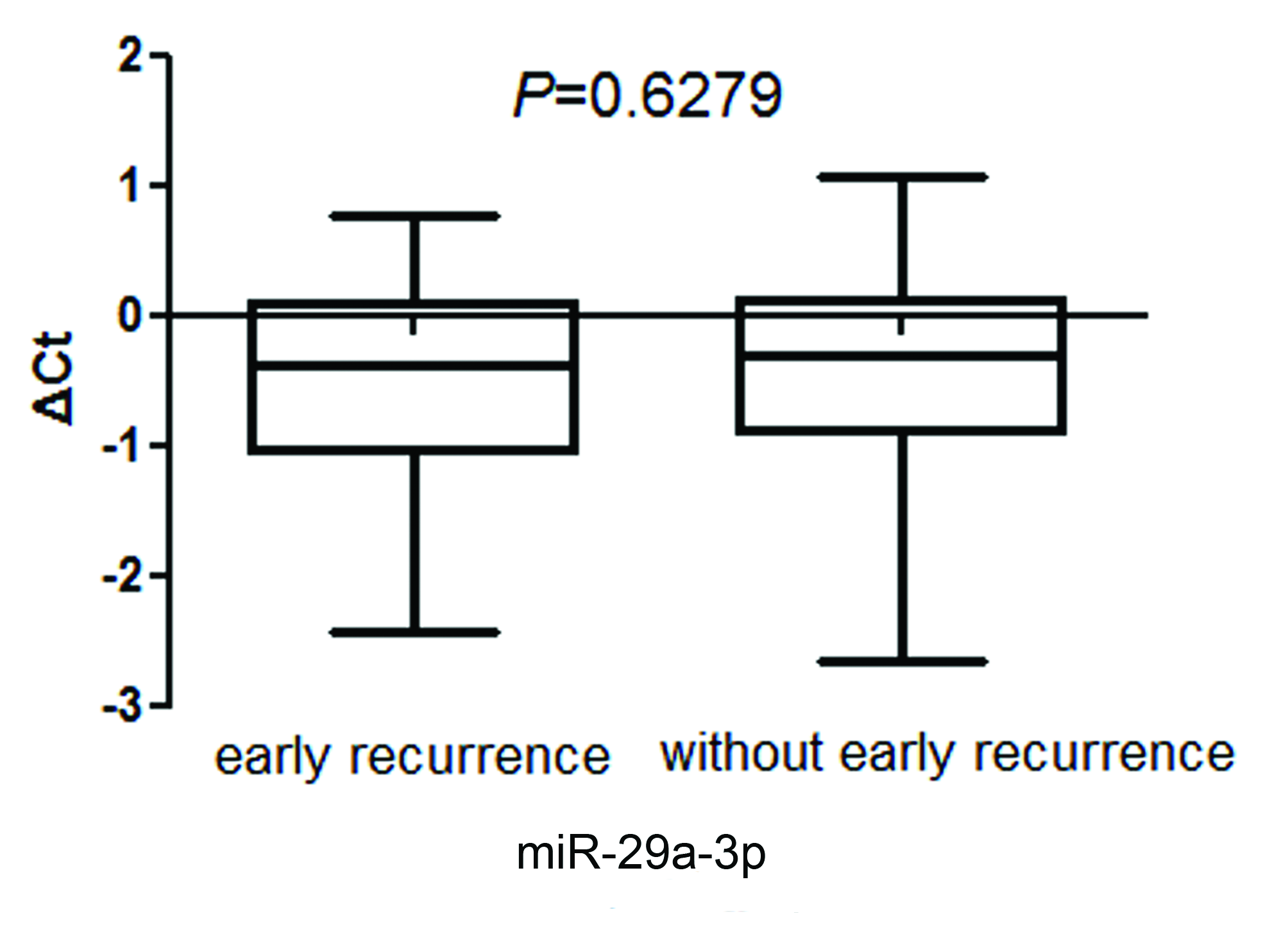

Supplement: Figure S6 — The association of miR-29a-3p level in microdissected HCC tissues with early tumor recurrence after HCC resection. Box and whiskers plots demonstrated that there was no significant difference of miR-29a-3p levels in HCC tissues was found between the patients with and without early recurrence in the training set (P = 0.6279). (TIF) [file pone.0052393.s006.tif]
