# Supplementary material for: MicroRNA-29a-5p Is a Novel Predictor for Early Recurrence of Hepatitis B Virus-Related Hepatocellular Carcinoma after Surgical Resection
Source: PLoS One. 2012 Dec 20;7(12):e52393. doi: 10.1371/journal.pone.0052393 (PMC3527523; doi:10.1371/journal.pone.0052393)
Supplement: Table S1 — Details of microRNAs and snRNAs enrolled in determination of suitable reference genes. (DOC) [file pone.0052393.s007.doc]

**Table S1. Details of microRNAs and snRNAs enrolled in determination of suitable reference genes**

| **Name** | **ABI Assay ID** | **Accession number** | **Chromosome Location** |
| --- | --- | --- | --- |
| U6 | 001973 | NR_004394** | 15q23 |
| RNU6B | 001093 | [NR_002752](http://microrna.sanger.ac.uk/cgi-bin/sequences/query.pl?terms=NR_002752)** | 10p13 |
| miR-103 | 000439 | [MIMAT000](http://microrna.sanger.ac.uk/cgi-bin/sequences/query.pl?terms=MIMAT0004495)0101* | 5q34 |
| miR-191 | 002299 | MIMAT0000440* | 3p21.31 |
| miR-25 | 000403 | MIMAT0000081* | 7q22.1 |
| miR-328 | 000543 | MIMAT0000752* | 16q22.1 |
| miR-374a | 000563 | MIMAT0000727* | Xq13.2 |
| miR-379 | 001138 | MIMAT0000733* | 14q32.31 |
| miR-26a-1* | 002443 | MIMAT0004499* | 3p22.2 |
| let-7a | 000377 | [MIMAT000](http://microrna.sanger.ac.uk/cgi-bin/sequences/query.pl?terms=MIMAT0002817)0062* | 9q22.32 |
| miR-625* | 002432 | MIMAT0004808* | 14q23.3 |
| miR-769-5p | 001998 | MIMAT0003886* | 19q13.32 |
| miR-889 | 002202 | MIMAT0004921* | 14q32.31 |

*MiRBase database accession number **Entrez gene ID.
