# Supplementary material for: MicroRNA-29a-5p Is a Novel Predictor for Early Recurrence of Hepatitis B Virus-Related Hepatocellular Carcinoma after Surgical Resection
Source: PLoS One. 2012 Dec 20;7(12):e52393. doi: 10.1371/journal.pone.0052393 (PMC3527523; doi:10.1371/journal.pone.0052393)
Supplement: Table S2 — Stability value and ranking of reference genes based on NormFinder. (DOC) [file pone.0052393.s008.doc]

**Table S2. -** Stability value and ranking of reference genes based on NormFinder

| **Rank** | **NormFinder** | |
| --- | --- | --- |
|  | **Gene** | **Stability** |
| Best combination | miR-103&mir-25 | 0.001 |
| 1 | miR-103 | 0.001 |
| 2 | miR-25 | 0.001 |
| 3 | miR-328 | 0.001 |
| 4 | miR-191 | 0.002 |
| 5 | let-7a | 0.002 |
| 6 | RNU6B | 0.002 |
| 7 | U6 | 0.003 |
