# Supplementary material for: MicroRNA-29a-5p Is a Novel Predictor for Early Recurrence of Hepatitis B Virus-Related Hepatocellular Carcinoma after Surgical Resection
Source: PLoS One. 2012 Dec 20;7(12):e52393. doi: 10.1371/journal.pone.0052393 (PMC3527523; doi:10.1371/journal.pone.0052393)
Supplement: Table S3 — Thirty-seven miRNAs identified that might be related to HCC recurrence after operation in this study. (DOC) [file pone.0052393.s009.doc]

**Table S3. Thirty-seven miRNAs identified that might be related to HCC recurrence after operation in this study**

| **Name** | **ABI Assay ID** | **Accession number** | **Chromosome Location** |
| --- | --- | --- | --- |
| miR-107^†^ | 000443 | MIMAT0000104 | 10q23.31 |
| miR-216a^†^ | 002220 | MIMAT0000273 | 2p16.1 |
| miR-517a^†^ | 002402 | MIMAT0002852 | 19p13.42 |
| miR-196b^†^ | 002215 | MIMAT0001080 | 7p15.2 |
| miR-210^†‡^ | 000512 | MIMAT0000267 | 11p15.5 |
| miR-98^†^ | 000577 | MIMAT0000096 | Xp11.22 |
| miR-135a^†^ | 000460 | MIMAT0000428 | 3p21.1 |
| miR-22^†‡^ | 000398 | MIMAT0000077 | 17p13.3 |
| miR-15b^†^ | 000390 | MIMAT0000417 | 3q25.33 |
| miR-505^†^ | 002089 | MIMAT0002876 | Xq27.1 |
| miR-518e^†^ | 002395 | MIMAT0002861 | 19q13.42 |
| miR-518f^†^ | 002388 | MIMAT0002842 | 19q13.42 |
| miR-522^†^ | 002413 | MIMAT0002868 | 19q13.42 |
| miR-519a^†^ | 002415 | MIMAT0002869 | 19q13.42 |
| miR-518b^†^ | 001156 | MIMAT0002844 | 19q13.42 |
| miR-10b^†^ | 002218 | MIMAT0000254 | 2q31.1 |
| miR-193b*^†‡^ | 002366 | MIMAT0004767 | 16p13.12 |
| miR-409-5p^†^ | 002331 | MIMAT0001638 | 14q32.31 |
| miR-215^†‡^ | 000518 | MIMAT0000272 | 1q41 |
| miR-217^†^ | 002337 | MIMAT0000274 | 2p16.1 |
| miR-486-5p^†‡^ | 001278 | MIMAT0002177 | 8p11.21 |
| miR-519d^†^ | 002403 | MIMAT0002853 | 19q13.42 |
| miR-643^†‡^ | 001594 | MIMAT0003313 | 19q13.41 |
| miR-483-5p^†^ | 002338 | MIMAT0004761 | 11p15.5 |
| miR-27b*^†^ | 002174 | MIMAT0004588 | 9q22.32 |
| miR-181c^†‡^ | 000482 | MIMAT0000258 | 19p13.13 |
| miR-204^†^ | 000508 | MIMAT0000265 | 9q21.12 |
| miR-29c^†^ | 000587 | MIMAT0000681 | 1q32.2 |
| miR-34c-5p^†^ | 000428 | MIMAT0000686 | 11q23.1 |
| miR-142-5p^†^ | 002248 | MIMAT0000433 | 17q22 |
| miR-766^‡^ | 001986 | MIMAT0003888 | Xq24 |
| miR-200a*^‡^ | 001011 | MIMAT0001620 | 1p36.33 |
| miR-10b*^‡^ | 002315 | MIMAT0004556 | 2q31.1 |
| miR-139-3p^‡^ | 002313 | MIMAT0004552 | 11q13.4 |
| miR-424*^‡^ | 002309 | MIMAT0004749 | Xq26.3 |
| miR-409-3p^‡^ | 002332 | MIMAT0001639 | 14q32.31 |
| miR-29a-5p^†^ | 002447 | MIMAT0004503 | 7q32.3 |

Accession number: miRBase database accession number.

†miRNAs that were identified differentially expressed in microdissected tumorous tissues by Taqman low density array

‡ miRNAs that were identified differentially expressed in microdissected non-tumorous liver tissues by Taqman low density array
